# An observational study of quality of motion in the aging cervical spine: sequence of segmental contributions in dynamic fluoroscopy recordings

**DOI:** 10.1186/s12891-024-07423-z

**Published:** 2024-04-25

**Authors:** Valérie N. E. Schuermans, Anouk Y. J. M. Smeets, Alexander Breen, Jonathan Branney, Inez Curfs, Henk van Santbrink, Toon F. M. Boselie

**Affiliations:** 1 Department of Neurosurgery, Maastricht University Medical Center, P. Debyelaan 25, 6229 HX Maastricht, The Netherlands; 2 Deptartment of Neurosurgery, Zuyderland Medical Center, Heerlen, the Netherlands; 3 CAPHRI School for Public Health and Primary Care, Maastricht University, Maastricht, the Netherlands; 4 College of Engineering, Mathematics and Physical Sciences, University of Exeter, Exeter, UK; 5 Faculty of Health and Social Sciences, Bournemouth University, Bournemouth, UK; 6 Deptartment of Orthopaedic Surgery and Traumatology, Zuyderland Medical Center, Heerlen, the Netherlands

**Keywords:** Cervical spine, Physiological motion, Aging, Range of motion, Sequence of segmental Contributions

## Abstract

**Background:**

The term ‘physiological motion of the spine’ is commonly used although no proper definition exists. Previous work has revealed a consistent sequence of cervical segmental contributions in 80–90% of young healthy individuals. Age has been shown to be associated with a decreased quantity of motion. Therefore, it is of interest to study whether this sequence persists throughout aging.

The aim of this prospective cohort study is to investigate if the consistent sequence of cervical segmental contributions in young asymptomatic individuals remains present in elderly asymptomatic individuals.

**Methods:**

In this prospective cohort study, dynamic flexion to extension cinematographic recordings of the cervical spine were made in asymptomatic individuals aged 55–70 years old. Individuals without neck pain and without severe degenerative changes were included. Two recordings were made in each individual with a 2-to-4-week interval (T1 and T2). Segmental rotation of each individual segment between C4 and C7 was calculated to determine the sequence of segmental contributions. Secondary outcomes were segmental range of motion (sRoM) and sagittal alignment.

**Results:**

Ten individuals, with an average age of 61 years, were included. The predefined consistent sequence of segmental contributions was found in 10% of the individuals at T1 and 0% at T2. sRoM and total range of motion (tRoM) were low in all participants. There was no statistically significant correlation between sagittal alignment, degeneration and sRoM in the respective segments, nor between cervical lordosis and tRoM.

**Conclusions:**

This study shows that aging is associated with loss of the consistent motion pattern that was observed in young asymptomatic individuals. The altered contribution of the cervical segments during extension did not appear to be correlated to the degree of degeneration or sagittal alignment.

Trial registration

clinicaltrials.gov NCT04222777, registered 10.01.2020.

**Supplementary Information:**

The online version contains supplementary material available at 10.1186/s12891-024-07423-z.

## Background

The term ‘physiological motion of the spine’ is commonly used, Although no proper definition exists [1–4]. This is mainly because the analysis of spinal motion remains a challenge in several aspects

Spinal motion is often investigated in terms of segmental range of motion (sRoM) in the sagittal plane [5, 6]. sRoM is measured by the amount of sagittal rotation in a segment between maximal flexion and maximal extension of the entire cervical spine. There are several methods for measuring and calculating sRoM, but they are often limited by high intra- and interindividual variability [1, 6–9]. Consequently, the ‘normal’ ranges that have previously been defined in healthy individuals are highly variable [10–13]. Despite the use of more precise automated measurements, the reported sRoM in individuals remains variable [14, 15]. Van Mameren et al. described several essential insights concerning the value of sRoM [6]. First, maximum sRoM can often not be determined by comparing full flexion to full extension, but can be present at another moment throughout the motion path. Second, sRoM differs depending on execution of the motion from flexion to extension or the other way around. Possibly due to different muscle activation during flexion and extension. Third, sRoM is time dependent, meaning that outcomes are not consistent when measured repeatedly, which was also confirmed by Bogduk et al. [5]. Therefore, total RoM (tRoM) and sRoM are unsuitable to be used as a parameter to define physiological motion in individuals.

To counter this problem, dynamic fluoroscopy recordings of flexion–extension movements have been analyzed. These revealed that the pattern of motion of the cervical spine during both flexion and extension was complex and counter-intuitive [6]. When investigating RoM in static radiographs, one assumes that maximum rotation is reached in each segment at the end of these movements. As previously observed by Boselie et al. in dynamic cinematographic recordings, this is not always the case. The maximum contribution of rotation varies per segment throughout the movement, and rarely occurs at the maximum extension or flexion [16].

**Fig. 1:**
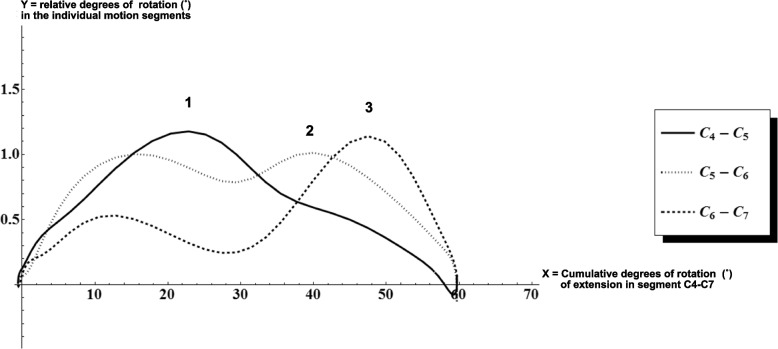
Example graph depicting relative rotation in motion segments in the lower cervical spine during extension movement in C4-C7, in an asymptomatic subject. Sagittal rotation (‘Rotation’, y-axis) of the individual motion segments of the lower cervical spine, set against cumulative sagittal rotation in the segment C4-C7 (i.e. the amount of extension in block C4-C7). The y-axis represents sagittal rotation (in degrees) of the individual motion segments of the lower cervical spine, while on the x-axis the cumulative sagittal rotation (in degrees) in C4-C7 is displayed. Peaks in the graphs depict maximum contributions of that motion segment. During the end of the extension movement the peak of C4-C5 (**1**) is followed by a peak in C5-C6 (**2**), and then C6-C7 (**3**) again. This last phase [1] is commonly found in extension in asymptomatic individuals (85%). Only the order of the peaks is important, not the height of the peaks on itself, except for very small peaks with a rotation lower than 0.3, which are deemed insignificant. In many cases there is a peak in C4-C5 at the end of extension, which is much smaller than the peak at (**1**), this is considered normal

In a previous study by our group, the sequence of segmental contributions during extension from C4 to C7 has been proposed as a more consistent parameter of cervical spine motion [5, 17]. The various motion segments are contributing at different moments throughout the extension movement. A consistent pattern was observed in 80–90% of 20 asymptomatic individuals during extension. The sequence of segmental contributions evolves from cranial to caudal [18–20]. The first peak was found in C4-C5, followed by C5-C6 and finally in C6-C7 (Fig. 1). This pattern was observed in individuals with an average age of 23 years (± 2.6). Age has been shown to be associated with a 0.11˚ decrease of sRoM per year, which means 5˚ decrease in motion of the entire subaxial cervical spine every 10 years of aging [21, 22]. Trott et al. described a similar significant decrease of sRoM in flexion/extension with age, especially when comparing 20 to 30-year-olds [23]. Particularly, a comparison between individuals aged 20–30 to 30–40 revealed a decrease from an average of 57.5˚ to 46.8˚ in flexion, and from 76.1˚ to 64.8˚ in extension, respectively. Moreover, they observed that all primary movements of the cervical spine are influenced throughout aging.

Despite the cervical spine kinematics research that has been conducted in the past decades, a clear definition of physiological motion is lacking due to the high variability of measured parameters. The previously identified consistent sequence in the lower cervical spine (C4-C7) was only investigated in young asymptomatic individuals. It is not known if this motion pattern persists with increasing age. A better fundamental knowledge of cervical spine motion is needed to gain understanding in the role of motion in the development of pathology and treatments.

Therefore, this study aims to investigate whether the previously described consistent sequence of segmental contributions in the lower cervical spine during extension, which is found in young asymptomatic individuals, is also present in asymptomatic elderly.

## Methods

### Study design

This is a fundamental research project in which asymptomatic elderly individuals without severe degenerative changes in the cervical spine were included. The study was approved by the medical ethical committee of the Zuyderland Medical Center (Z2019087) and registered before the start of the study on 10.01.2020 (NCT04222777). An external validation was performed by analyzing recordings made in the Center of Biomechanics Research lab, AECC university college, Bournemouth, UK [24]. Informed consent was obtained from all participating individuals before study participation.

### Aim

The primary objective is to investigate whether the previously defined consistent sequence of segmental contributions in extension from C4-C7 in asymptomatic young individuals, is also present in an asymptomatic elderly population. Secondary objectives will be to determine the sRoM of C4-C5, C5-C6, and C6-C7 for comparability with other literature, and to investigate the influence of cervical lordosis and segmental degeneration on the presence of this motion pattern. An external validation will be performed in an independent study population from Bournemouth AECC, to verify the findings of the current study.

### In- and exclusion criteria

Male and female individuals between 55 and 70 years of age, capable of actively performing flexion and extension movements of the cervical spine without pain or other complaints related to neck pain, as defined by a score of 4 or less points on the Neck Disability Index (NDI). Individuals with symptoms of radiculopathy and/or myelopathy were excluded. Other exclusion criteria were; an active infection, previous or actual tumorous processes in the cervical region, previous radiotherapy in the cervical region or not able to speak the Dutch language.

A lateral X-ray of the cervical spine was made after inclusion to determine the Kellgren score (KS) [25]. The KS was independently determined by two neurosurgeons. Individuals with as a KS of four were excluded [25, 26].

The same in- and exclusion criteria were applied to a database of asymptomatic individuals from Bournemouth AECC [27].

### Image acquisition

For the Zuyderland Medical Center population, all cinematographic recordings were made at the same location, following the same protocol. Individuals were seated on a height-adjustable crutch, without support of the neck, shoulders and head. They were asked to move their head from maximal flexion to maximal extension without moving the upper part of their body in a period of 10 s, using a metronome. Movement of the cervical spine was as fluent as possible to prevent sudden large rotations and translations between consecutive frames. Fluoroscopic recordings were made with a Philips Allura Xper FD20 X-ray system, capturing frames of 1024 × 1024 pixels, at 10 frames per second. Two recordings were made in each individual, with a two-to-four-week interval.

For the Bournemouth AECC study population, the protocol was comparable, only here individuals performed guided movement instead of an unguided movement. Details of the image acquisition for the external validation group have been previously published [28].

No treatments or interventions took place within the time interval between the two timepoints (T1 and T2) in both populations.

### Data analysis

**Fig. 2:**
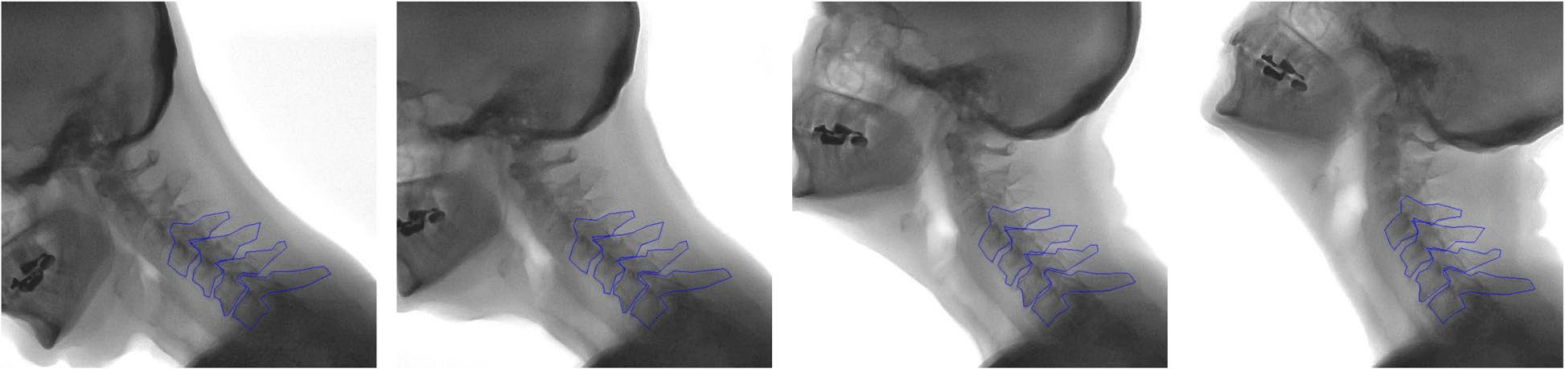
Separate frames of an extension cinematographic recording. Four out of 52 frames of the movement are displayed, starting from maximum flexion (left) to maximum extension (right). Annotation of the vertebrae C4 to C7 in each separate frame with manual corrections are displayed. Relative rotations of the segments are calculated based on these annotations

All images were analyzed using computer software that uses an image recognition algorithm to follow motion of the vertebrae during complete flexion and extension, developed in Wolfram Mathematica (Wolfram Research, Inc., Version 9.0, Champaign, IL). This method has been previously described and validated [6, 20]. All contours were manually checked, and if necessary adjusted, to fit the corresponding vertebra in every frame of the recording (Fig. 2). The investigation focused solely on the pattern observed in the lower cervical spine, as the objective was to compare the sequential contributions of segments in this particular population with the established sequence observed in young asymptomatic individuals. Vertebrae C0-C3 were not analyzed, primarily due to the highly time-consuming nature of this type of analysis.

Segmental rotation between each pair of successive frames of each individual motion segment within C4 to C7 was plotted against the cumulative rotation in block C4 to C7 in order to describe the sequence of segmental contributions (SSC) during extension. These graphs were made and analyzed for each individual to determine if a consistent sequence of segmental contributions is present or absent. KS and sagittal alignment were determined on an X-ray taken in neutral position. Sagittal alignment was determined according to Cobb’s angle from C2 to C7, and from the upper endplate to the lower endplate for each individually analyzed segment.

### Sample size calculation

Sample size calculation was based on the previous study by our group using the same method of cinematographic recordings to analyze motion patterns of the cervical spine [20, 29]. Ten individuals were required and if for the first ten individuals two recordings were made with good quality, recruitment would be ended [6, 20].

### Statistical analyses

Recordings were acquired twice for each individual with an interval of two weeks in order to determine reproducibility and consistency of sequence of motion between two time points (T1 and T2). Presence of SCC pattern consistent with a younger population was tested for, comparing T1 against T2 using coefficient of variation and intra-individual standard deviation. Inter observer reproducibility and consistency was measured using two-way mixed average measurements intra-class correlation coefficients (ICC) for absolute agreement. To achieve this, across four cinematographic recordings, individual sRoM were analyzed by two researchers. No interim analysis was performed. Descriptive statistics were used to describe baseline characteristics and the primary outcome. A Spearman’s was used to investigate the correlations between degeneration (Kellgren score, KS), sagittal alignment (Cobb’s angle, C2-C7) and segmental range of motion (sRoM) as secondary outcomes. For the external validation a convenience sample was taken.

## Results

### Population

A total of 10 individuals were included. Baseline characteristics are outlined in Table 1. The average age of the study population was 61 years. Overall, degeneration according to the KS was low. Three individuals showed relatively higher degrees of degeneration (individuals 3, 4 and 6). The ICC of 12 segments was 0.843 on average [Appendix 1].

**Table 1:** Baseline characteristics of study population. M = male, F = female, NDI = Neck Disability Index, KS = Kellgren’s Score, SD = standard deviation

Individual	Sex (M/F)	Age	NDI (%)	KS C4-C5	KS C5-C6	KS C6-C7	‘Normal’ sequence ( ±)	
							**T1**	**T2**
1	M	58	0	1	1	1	-	-
2	F	55	3 (6%)	1	1	1	-	-
3	F	67	2 (4%)	1	2	2	-	-
4	M	67	3 (6%)	3	3	3	-	-
5	F	64	0	1	1	2	-	-
6	M	63	0	3	3	2	-	-
7	M	60	2 (4%)	2	1	1	-	-
8	M	57	0	1	1	1	-	-
9	F	59	0	1	1	3	-	-
10	M	61	0	1	1	2	+	-
**Average (SD)**	**6:4 M:F**	**61 [57;67]**	**1 [0;3]**	**1.5 [1;3]**	**1.5 [1;3]**	**1.8 [1;3]**	**10%**	**0%**

**Fig. 3:**
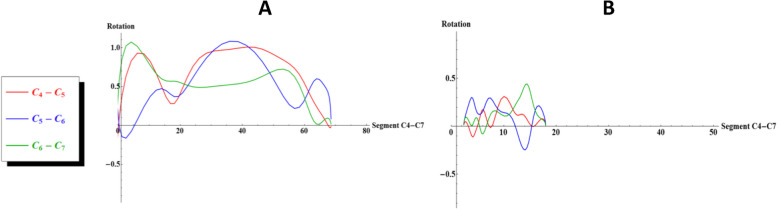
The graphs illustrate sagittal rotation in segments of the lower cervical spine (block C4-C7) during the extension of the entire cervical spine. Sagittal rotation (‘Rotation’, y-axis) of the individual motion segments of the lower cervical spine, set against cumulative sagittal rotation in the segment C4-C7 (i.e. the amount of extension in block C4-C7). The y-axis represents sagittal rotation (in degrees) of the individual motion segments of the lower cervical spine, while on the x-axis the cumulative sagittal rotation (in degrees) in C4-C7 is displayed. In Fig. 3A, an individual is depicted with observable motion, although no consistent pattern can be identified (S02, T1). In Fig. 3B, another individual is shown with minimal amount of cumulative motion, and the segments exhibit high variability in contributions, including instances of negative motion (S01, T2)

The predefined normal sequence of motion, C4-C5 contributing first to rotation, followed by C5-C6 and then C6-C7, was only found in one individual at T1 (10%) and in none of the individuals at T2 (0%). Figure 3 shows to representative graphs of two individuals. The complete overview of graphs of relative rotation patterns of each individual can be found in Appendix 2. Subgroup analyses were not conducted due to the lack of a consistent sequence.

### Secondary outcomes

SRoM was determined for each individual segment from C4 to C7 at T1 and T2 [Table 2]. There was no significant correlation between tRoM and KS per segment, C4-C5 (p = 0.757), for C5-C6 (*p* = 0.318) and C6-C7 (*p* = 0.887). Spearman correlation coefficients did not show a statistically significant correlation between KS and sROM for C4-C5 (*p* = 0.789), for C5-C6 (*p *= 0.256) and C6-C7 (*p* = 0.446).

**Table 2:** Ranges of motion per individual per segment expressed in degrees, for both T1 and T2. tROM = total range of motion from C4-C7, sROM = segmental range of motion, SD = standard deviation

**Individual**	**T1**				**T2**			
	**tROM (˚)** **C4-C7**	**sROM (˚)** **C4-C5**	**sROM (˚)** **C5-C6**	**sROM (˚)** **C6-C7**	**tROM (˚)** **C4-C7**	**sROM (˚)** **C4-C5**	**sROM (˚)** **C5-C6**	**sROM (˚)** **C6-C7**
1	23.2	14.7	0.2	8.3	15.3	3.6	5.0	6.8
2	55.8	23.9	20.9	11.0	53.5	14.5	17.5	21.6
3	13.1	6.5	2.7	4.0	10.8	4.4	2.9	3.5
4	25.2	4.9	0.2	20.2	8.1	5.9	1.0	1.1
5	21.1	13.1	4.7	3.3	19.4	12.5	3.5	3.4
6	24.9	9.7	8.2	7.0	22.1	11.1	6.4	4.5
7	15.5	10.2	4.7	0.6	23.9	14.5	5.7	3.8
8	16.4	9.2	7.3	0.2	13.9	6.5	4.8	2.6
9	27.4	9.1	14.7	3.7	22.4	11.5	8.6	2.3
10	35.5	10.2	15.3	10.1	33.6	8.9	15.2	9.5
**Average (SD)**	**25.8 [15.5;55.8]**	**11.1 [4.9;23.9]**	**7.9 [0.2;20.9]**	**5.8 [0.2;20.2]**	**22.3 [8.1;53.3]**	**9.3 [3.6;14.5]**	**7.1 [1.0;17.5]**	**5.9 [1.1;21.6]**

Observed sagittal alignment was highly variable between individuals [Table 3]. A segmental kyphosis was observed in segments with a KS of 2 or 3 in individuals 3 and 4, although this was also observed in individual 2 who had limited degeneration (KS of 1). Individual 6 showed a straight spine, with kyphosis in segment C6-C7. Spearman correlation coefficients did not show a statistically significant correlation between sagittal alignment, KS and sRoM in the respective segments, for C4-C5 (*p* = 0.701), for C5-C6 (*p* = 0.774) and C6-C7 (*p* = 0.056), nor between cervical lordosis and tRoM (*p* = 0,713).

**Table 3:** Sagittal alignment per individual measured according to Cobb’s angle. SA = Sagittal alignment, SD = Standard deviation

Individual	SA (˚) C2-C7	SA (˚) C4-C5	SA (˚) C5-C6	SA (˚) C6-C7
1	23	6	2	3
2	-4	-11	-11	-9
3	2	-5	-1	-4
4	-4	-1	-7	4
5	17	1	-3	6
6	8	2	3	-3
7	35	4	13	12
8	31	4	3	4
9	19	-2	3	4
10	21	2	1	1
**Average ± SD**	**14.8 [-4;35]**	**0.00 [-11;4]**	**0.30 [-11;13]**	**1.8 [-9;12]**

### External validation

Three out of 33 asymptomatic individuals at AECC Bournemouth fulfilled our inclusion criteria, and their recordings of T1 and T2 were analyzed with the same method as the study population [Table 4]. Findings were similar to those in our own population; an absence of the normal sequence that was found in young asymptomatic volunteers and a significantly lower sRoM and tRoM in elderly (Schuermans VNE, Breen A, Branney J, Smeets AYJM, van Santbrink H, Boselie TFM: Cross-validation of two independent methods to analyze the sequence of segmental contributions in the cervical spine in extension cineradiographic recordings, unpublished). It should be noted that these participants started the motion in neutral position and then moved to maximum extension. Therefore, sRoM cannot be directly compared between the two groups.

**Table 4:** Data from the 3 patients in the external validation group. Ranges of motion per individual per segment expressed in degrees, for both T1 and T2. Important to note that this only concerns the second half of extension in this group. tROM = total range of motion from C4-C7, sROM = segmental range of motion, SD = standard deviation

**External validation**	**T1**				**T2**				**Presence of normal sequence ( ±)**	
	tROM (˚) **C4-C7**	**sROM (˚)** **C4-C5**	**sROM (˚)** **C5-C6**	**sROM (˚)** **C6-C7**	**tROM (˚)** **C4-C7**	**sROM (˚)** **C4-C5**	**sROM (˚)** **C5-C6**	**sROM (˚)** **C6-C7**	**T1**	**T2**
E1	11.2	7.4	1.00	2.8	12.4	6.2	3.3	2.8	-	-
E2	10.6	4.2	4.4	2.0	10.3	4.1	5.4	0.7	-	-
E3	8.2	2.2	2.5	3.5	12.6	4.0	4.2	4.3	-	-
**Average ± SD**	10.0 [8.2;11.2]	4.6 [2.2;7.4]	2.6 [1;4.4]	2.8 [2;3.5]	11.8 [10.3[12.6]	4.8 [4;6.2]	4.3 [3.3;5.4]	2.6 [0.7;4.3]	0%	0%

## Discussion

The key finding of this study is that aging is associated with a loss of consistent motion patterns within the cervical spine. Only one individual exhibited a motion pattern consistent with that found in 80–90% of young asymptomatic individuals. Importantly, these altered segmental contributions during extension were evident in elderly individuals without severe radiological degeneration or neck pain, indicating the involvement of an alternative mechanism. This novel fundamental insight has the potential to reshape our understanding of "physiological" motion in the cervical spine. We show that aging is not only associated with a decrease in quantity of motion, but also with a change in quality of motion.

Since this study involved elderly individuals with limited degenerative changes, the absence of a consistent pattern of segmental contributions as found in younger individuals, can thus not be attributed to radiological degeneration alone. Moreover, the degree of radiological degeneration does not correlate with the measured sRoM and sagittal alignment, suggesting that other factors contribute to the observed changes. The large variation of sROM between T1 and T2 is in line with previous studies that state sROM to be an unreliable outcome measure which varies over time [5]. A lack of correlation between sRoM, tRoM and sagittal alignment in healthy asymptomatic individuals was also reported by Inoue et al. [30]. Interestingly, the sRoM observed in each segment of the cervical spine in this study was lower than the ranges previously reported in literature, which defined normal ranges [5]. However, it is important to note that these previously established ‘normal’ ranges were derived from younger populations [5].

Miyazaki et al. conducted an magnetic resonance imaging (MRI) study to investigate the relationship between sagittal alignment and kinematics in the cervical spine [31]. They found that in individuals with straight cervical spines, there was a tendency for the upper levels (C2-C3, C3-C4) to have an increased contribution to sagittal rotation, while the middle (C4-C5, C5-C6) and lower levels (C6-C7) exhibited a decreased contribution. This change in alignment was associated with greater disc degeneration at the upper levels, but not at the middle and lower levels. These findings are consistent with our observations, where we identified altered motion patterns and reduced sRoM in the lower levels, which were not correlated with radiological degeneration.

It is worth noting that the average cervical lordosis measured from C2-C7 in our elderly study population is 14.8˚, which is considerably lower than the "normal" average of 34˚ [range 16.5˚-66˚] reported in young individuals [32]. This observation of decreased lordosis in those older than 60 years was also reported by Park et al. [33]. It contradicts the common notion that during aging there is a tendency towards kyphotic deformity of the whole spine [34]. Possibly, variations in positioning of individuals during the acquisition of lateral radiographs may partly explain the differences in findings.

The disparity in motion patterns observed could potentially be attributed to postural factors, as was previously proposed by Edmondston et al. [35]. In their study, they examined the impact of the initial position on the pattern of primary and coupled movements in a young and asymptomatic population. They found that motion patterns varied depending on the starting position. However, in our study population, the extension movement originated from full flexion, while the external validation group began from a neutral position. Despite this difference in starting position, similar findings were obtained. Similar findings were reported by Park et al., who observed no changes in sRoM when comparing maximum flexion and extension to the neutral position but noted changes during flexion and extension specifically in the lower cervical spine [33].

Variations in patterns could potentially be attributed to differences in muscle recruitment during active movements [36]. However, in the present study, free active motion and guided active motion did not influence the observed motion patterns. This finding is consistent with another study conducted by our research group, where asymptomatic younger individuals exhibited similar patterns of motion regardless of whether the motion was free or guided (Schuermans VNE, Breen A, Branney J, Smeets AYJM, van Santbrink H, Boselie TFM: Cross-validation of two independent methods to analyze the sequence of segmental contributions in the cervical spine in extension cineradiographic recordings, unpublished).

Several studies have reported differences in tRoM and sRoM between active and passive movements, implying a role of muscle activity in motion patterns [37, 38]. The inclusion of individuals with a low NDI mitigates the influence of myogenic strain on motion patterns in this study.

Sforza et al. have highlighted a significant difference of 130˚ tRoM in adolescents vs 116˚ in a mid-aged population, which appeared to be influenced by athletic activity and body weight [39]. Factors such as body composition, muscle thickness, and individual anatomical variations may impact the habitual motion of the cervical spine.

Another plausible explanation could be that the observed pattern becomes obscured due to the decrease in range of motiona, leading to a lower signal to noise ratio. As the peaks of segmental contributions become lower, the differences between these peaks may become smaller, potentially falling below the detection limit and resulting in an altered pattern. The reduction in RoM could be attributed to an increase in cervical spine rigidity, as ligamentous laxity typically decreases with age, rather than being solely influenced by osseous degeneration and alignment.

The findings of the present study suggest that while a consistent pattern may indeed exist, it gradually diminishes over time. While we propose several hypotheses to account for this observed decline in motion quality, it simultaneously introduces novel inquiries that necessitate additional exploration.

### Limitations

This study has some limitations to be considered when interpreting the results. First of all, the small sample size. However, a similar sample has previously proven to be sufficient to recognize a consistent motion pattern in a younger population. Gender, BMI, level of degeneration and sagittal alignment were not investigated as confounding factors, given the absence of a consistent sequence in any of the participants. Another aspect that was not investigated in this study are the fitness levels of included participants.

Only rotation in the sagittal plane was investigated. Analysis of translation in the sagittal or coronal plane, lateral flexion, and axial rotation are not evaluated. The instantaneous center of rotation was not included in this study as preliminary findings were highly variable. Further investigations could give more insight. While a consistent motion pattern was observed specifically during extension in segments C4-C7 in our younger, asymptomatic population, it has been suggested that the higher cervical vertebrae play a major role in flexion, while less in extension. The role of the upper cervical vertebrae might need further investigation. Possibly, more extensive analysis of all the segments is needed. Another aspect to consider is the threshold that we defined as ‘elderly’. We focused on 55 to 70 years old, which is arbitrary. It is possible that the measurement error increases as the RoM decreases, as very small rotations are measured, which might influence the findings of this study. An external validation was performed and the fact that the same findings are observed in an external group and with another method make the results of this study more reliable. Moreover, the same method found consistent patterns in younger individuals, which makes it unlikely that the method in this study fails (Schuermans VNE, Breen A, Branney J, Smeets AYJM, van Santbrink H, Boselie TFM: Cross-validation of two independent methods to analyze the sequence of segmental contributions in the cervical spine in extension cineradiographic recordings, unpublished).

## Conclusion

This study suggests that aging is linked to changes in motion patterns. The variations in the contribution of cervical segments during extension did not show correlation with the degree of degeneration or sagittal alignment. This highlights the necessity for a deeper understanding of cervical spine motion to grasp its role in pathology development and treatment strategies.

### Supplementary Information

**Supplementary Material 1.**

**Supplementary Material 2.**

## Data Availability

The datasets generated during and/or analyzed during the current study are available in the appendices. Additional information can be obtained from the corresponding author on reasonable request.

## Abbreviations

CDDD: Cervical degenerative disc disease
KS: Kellgren score
NDI: Neck disability index
RoM: Range of motion
SA: Sagittal alignment
sRoM: Segmental range of motion
tRoM: Total range of motion
