# Supplementary material for: An observational study of quality of motion in the aging cervical spine: sequence of segmental contributions in dynamic fluoroscopy recordings
Source: BMC Musculoskelet Disord. 2024 Apr 25;25:330. doi: 10.1186/s12891-024-07423-z (PMC11044387; doi:10.1186/s12891-024-07423-z)
Supplement: Supplementary file 1 — Supplementary Material 1. [file 12891_2024_7423_MOESM1_ESM.docx]

Appendix 1: Intraclass correlation coefficients of two individual observers per recordings.

| **P10** | **Cronbach’s alpha** | **Range** | **p-value** |
| --- | --- | --- | --- |
| C4-5 | 0.879 | 0.788-0.932 | <0.001 |
| C5-6 | 0.885 | 0.798-0.935 | <0.001 |
| C6-7 | 0.826 | 0.693-0.901 | <0.001 |
| **P09** | | | |
| C4-5 | 0.907 | 0.836-0.947 | <0.001 |
| C5-6 | 0.535 | 0.180-0.736 | 0.004 |
| C6-7 | 0.481 | 0.086-0.706 | 0.012 |
| **RCT 09** | | | |
| C4-5 | 0.958 | 0.925-0.976 | 0.000 |
| C5-6 | 0.993 | 0.987-0.996 | 0.000 |
| C6-7 | 0.803 | 0.653-0.888 | <0.001 |
| **RCT 25** | | | |
| C4-5 | 0.975 | 0.956-0.986 | 0.000 |
| C5-6 | 0.983 | 0.971-0.991 | 0.000 |
| C6-7 | 0.895 | 0.815 = 0.941 | <0.001 |
| **Average = 0.843 [0.481-0.993]** | | | |
