# Supplementary material for: An observational study of quality of motion in the aging cervical spine: sequence of segmental contributions in dynamic fluoroscopy recordings
Source: BMC Musculoskelet Disord. 2024 Apr 25;25:330. doi: 10.1186/s12891-024-07423-z (PMC11044387; doi:10.1186/s12891-024-07423-z)

Legend

Graphs depicting sagittal rotation in segments in the lower cervical spine (block C4-C7) during extension of the entire cervical spine, in a healthy control. On the y-axis sagittal rotation between successive frames is shown. On the x-axis cumulative extension in block C4-C7 is shown. Peaks in the graphs depict maximum contributions of a segment in that phase of extension of the entire cervical spine.

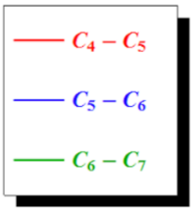

	T1	T2
S01	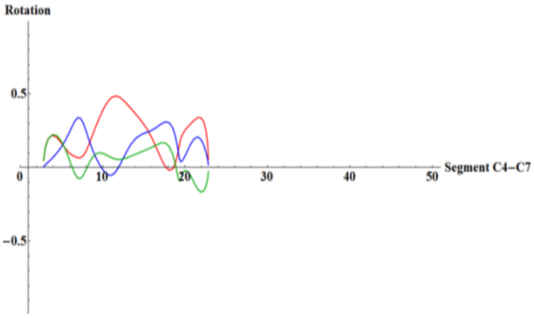	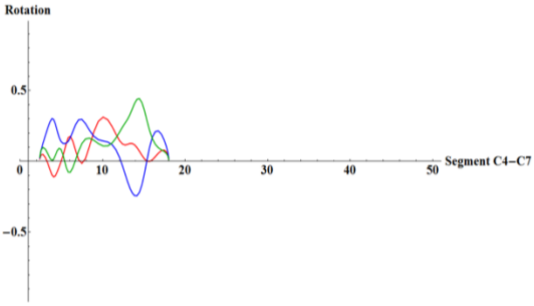
S02	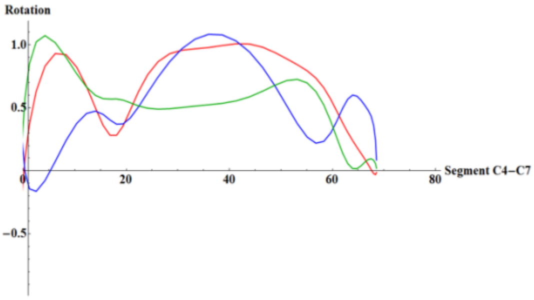	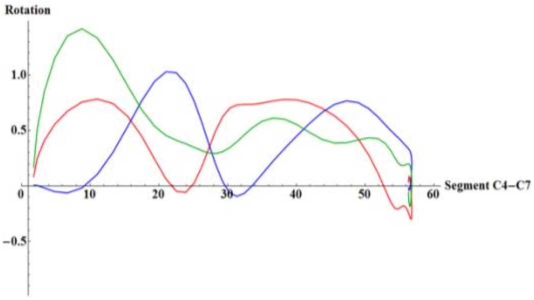
S03	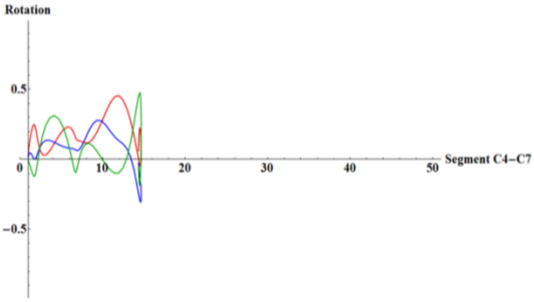	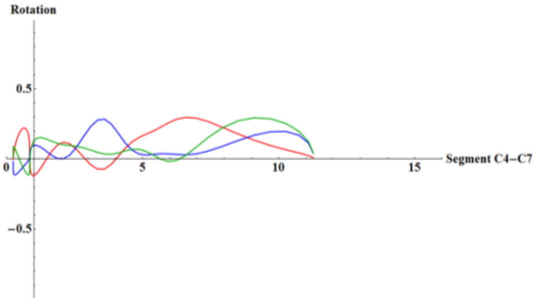
S04	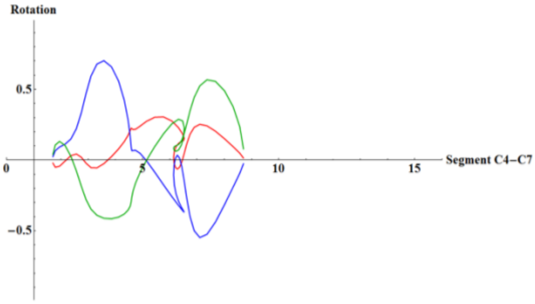	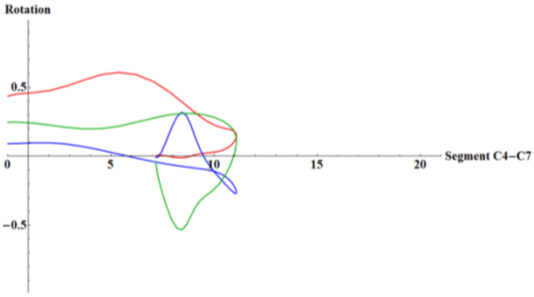

S05

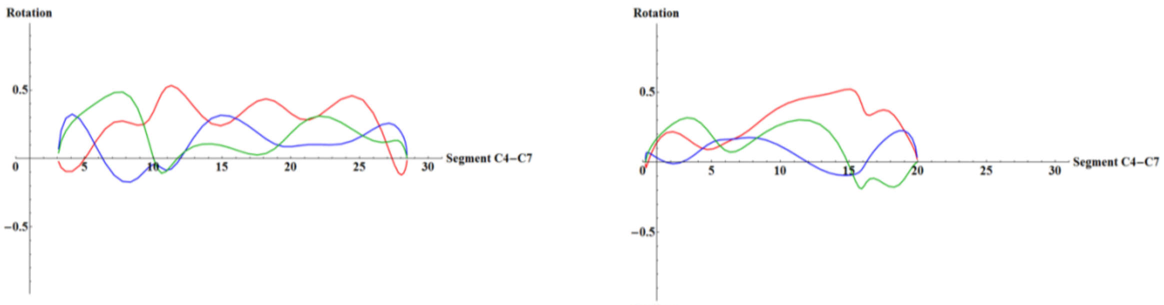

S06

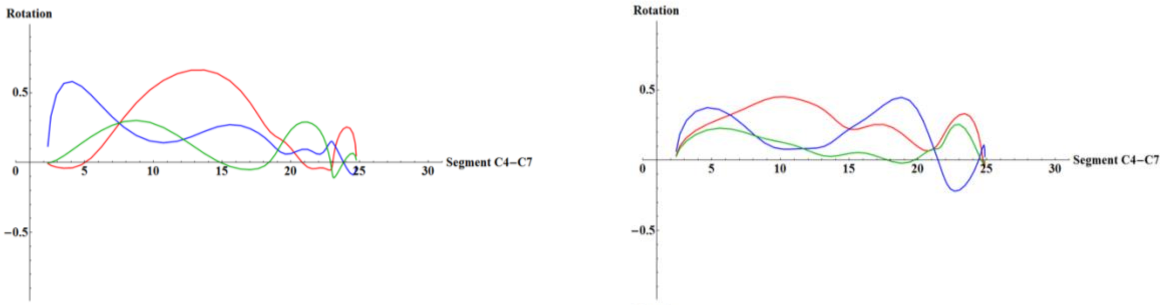

S07

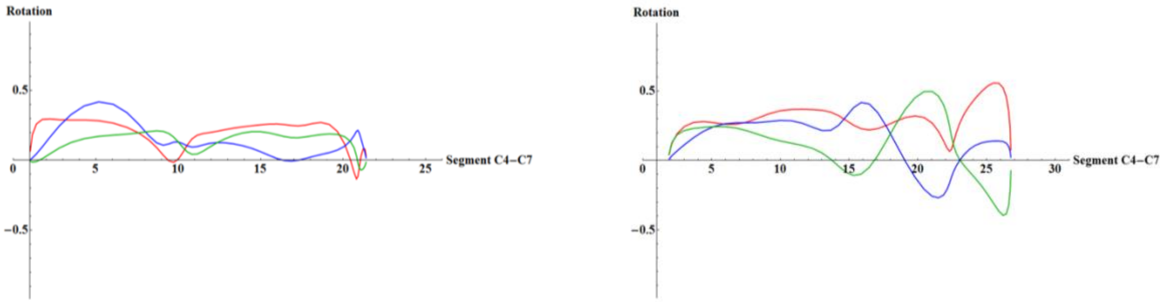

S08

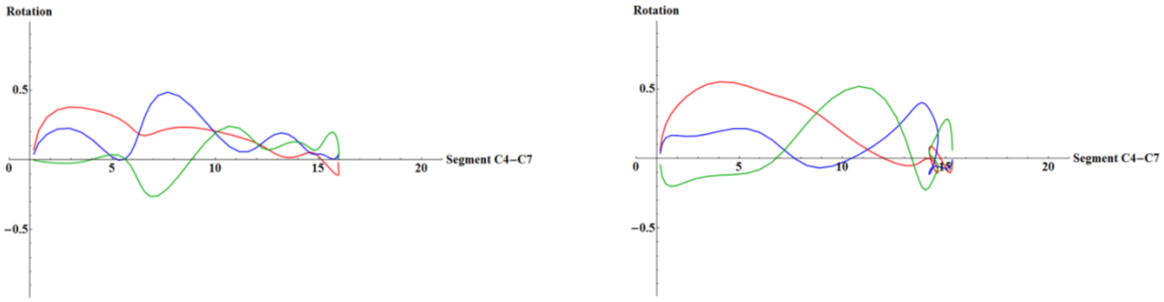

S09

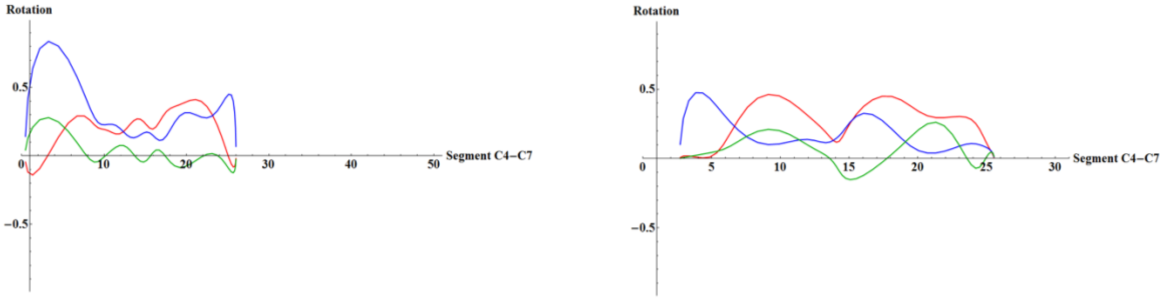

S10

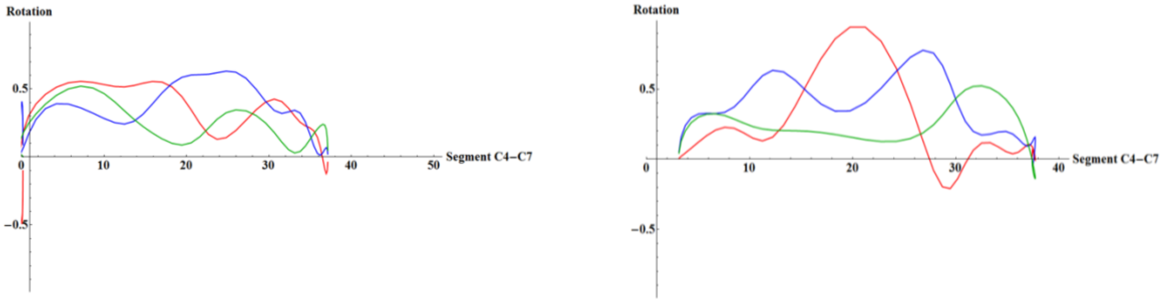

Supplement: Supplementary file 2 — Supplementary Material 2. [file 12891_2024_7423_MOESM2_ESM.pdf]
